# Fast Sleep Stage Classification Using Cascaded Support Vector Machines with Single-Channel EEG Signals

**DOI:** 10.3390/s22249914

**Published:** 2022-12-16

**Authors:** Dezhao Li, Yangtao Ruan, Fufu Zheng, Yan Su, Qiang Lin

**Affiliations:** 1 Zhejiang Provincial Key Laboratory of Quantum Precision Measurement, Collaborative Innovation Center for Information Technology in Biological and Medical Physics, College of Science, Zhejiang University of Technology, Hangzhou 310023, China; 2 School of Art, Zhejiang International Studies University, Hangzhou 310023, China

**Keywords:** single-channel EEG signals, sleep stage classification, cascaded support vector machine, nonlinear dynamics features, long-term monitor

## Abstract

Long-term sleep stage monitoring is very important for the diagnosis and treatment of insomnia. With the development of wearable electroencephalogram (EEG) devices, we developed a fast and accurate sleep stage classification method in this study with single-channel EEG signals for practical applications. The original sleep recordings were collected from the Sleep-EDF database. The wavelet threshold denoising (WTD) method and wavelet packet transformation (WPT) method were applied as signal preprocessing to extract six kinds of characteristic waves. With a comprehensive feature system including time, frequency, and nonlinear dynamics, we obtained the sleep stage classification results with different Support Vector Machine (SVM) models. We proposed a novel classification method based on cascaded SVM models with various features extracted from denoised EEG signals. To enhance the accuracy and generalization performance of this method, nonlinear dynamics features were taken into consideration. With nonlinear dynamics features included, the average classification accuracy was up to 88.11% using this method. In addition, with cascaded SVM models, the classification accuracy of the non-rapid eye movement sleep stage 1 (N1) was enhanced from 41.5% to 55.65% compared with the single SVM model, and the overall classification time for each epoch was less than 1.7 s. Moreover, we demonstrated that it was possible to apply this method for long-term sleep stage monitor applications.

## 1. Introduction

Sleep, which contributes to self-recovery, replenishing psychophysiological resources, and upholding the immune system, is a critical physiological activity of the human body. Recently, sleep health has been widely discussed due to its association with mortality, coronary artery disease [1], and impaired neurobehavioral performance [2,3]. Unfortunately, according to demographics, up to 24% of the adult population suffers from various sleep problems, including insomnia, obstructive sleep apnea syndrome, or a mere lack of sleep hygiene, and sleep therapy is urgently needed [4]. To provide effective diagnosis and treatments, long-term sleep monitoring and sleep stage classification are very necessary [5,6]. According to Rechtschaffen, A., and Kales, A.D. (R&K) rules [7] and the recently updated American Academy of Sleep Medicine (AASM) standard [8], sleep can be evaluated and classified into five different stages: wake, non-rapid eye movement sleep stages (1, 2, 3), and rapid eye movement, namely W, N1, N2, N3, and R. 

Currently, Polysomnography (PSG) is considered to be an effective method for sleep stage classification [9], but it has several obvious disadvantages. PSG is very cumbersome and needs to record multiple bio-signals of the patient simultaneously, including electromyogram (EMG), electrocardiogram (ECG), electroencephalogram (EEG), electrooculogram (EOG), blood oxygen saturation, etc. [10,11]. To carry out PSG, the patients are required to stay in a special sleep lab for at least one whole night. These requirements make this method costly and time-consuming, limiting its applications in fast and long-term sleep monitoring [12]. To overcome the above-mentioned shortcomings, one promising strategy using wearable electroencephalogram (EEG) signal-acquiring systems has been proposed for sleep stage classification [13,14], since EEG signals have different characteristics in different sleep stages. Based on this property, kinds of signal processing techniques have been applied to extract sleep-related feature information, including time-domain features [15], spectral features [16], time–frequency features [17], and nonlinear dynamics features [18]. What is more, to determine the sleep stage, several kinds of algorithms including K-means [19], Support Vector Machine (SVM) [20], Random Forest [21], Naive Bayes [22], Convolutional Neural Network (CNN), and Recurrent Neural Network (RNN) [23,24,25] were proposed. Sors, A. reported a one-dimensional Convolutional Network method for sleep stage classification, and the average accuracy was 87% [26]. Using EEG signal energy features and Recurrent Neural Network, Hsu et al. developed another system to classify five sleep stages, resulting in 87.2% average accuracy [27]. Most work focuses on classification accuracy but ignores the classification calculation time. Few studies discussed how to strike a balance between the high classification accuracy and time-consuming aspect. In addition, it was difficult to obtain an accuracy higher than 40% for the N1 stage with single-channel EEG signals. To meet the requirement for long-term sleep monitoring, an accurate and fast sleep stage classification method with single-channel EEG signals is highly desired.

To address the aforementioned challenges, based on cascaded SVM models we proposed a fast sleep stage classification method with single-channel EEG signals following the AASM rules. We applied the nonlinear dynamics features of EEG signals, which resulted in a more comprehensive feature system to improve the accuracy and generalization performance. Moreover, the classification speed with this method has also been evaluated. These results revealed that it would be very promising to use this method for practical long-term sleep stage monitoring in the future.

## 2. Materials and Methods

**Figure 1:**
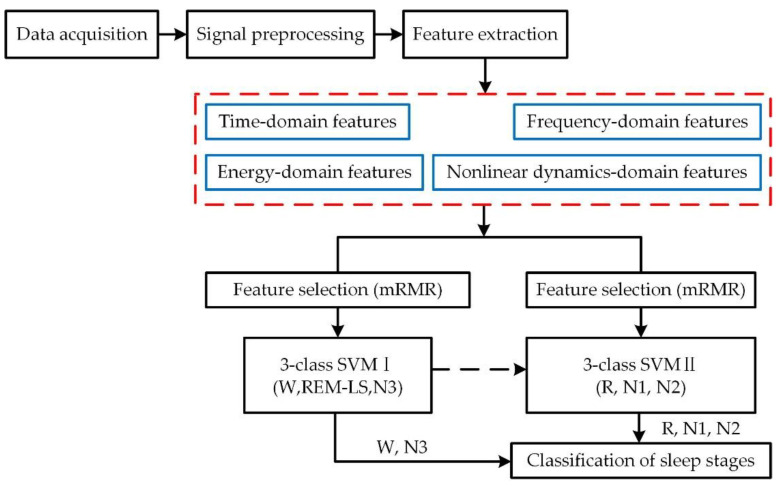
The sleep stage classification processing flow adopted in this paper.

The scheme of the sleep stage classification process adopted in this study is described in Figure 1, including data acquisition from sleep recordings, signal preprocessing, feature extraction, feature selection, and classification. The original sleep recordings were collected from the Sleep-EDF database [28,29], and we chose the data from the Fpz-Cz channel to analyze [30]. The wavelet threshold denoising (WTD) method and wavelet packet transformation (WPT) method were applied in the process of signal preprocessing to obtain six kinds of characteristic waves. After this, a comprehensive feature system with time, frequency, and nonlinear dynamics domains was made up. Subsequently, the minimum redundancy maximum relevance (mRMR) algorithm was used to select the most effective features. Finally, we obtained the cascaded SVM models with these selected features as the inputs. The final classification results were a combination of the calculation results from the two SVM models.

### 2.1. Data Collection

The EEG sleep recordings used in this study were obtained from the Sleep-EDF database, which is publicly available from PhysioBank directly [28,29]. We collected eight different sleep data sets from healthy people (SC4001, SC4011, SC4021, SC4051, SC4062, SC4102, SC4112, and SC4122) aged from 21 to 35. Originally, the sleep recordings had three kinds of signals, which were the horizontal electrooculogram (EOG) and EEG signals from Fpz-Cz and Pz-Oz channels. All these signals were recorded with the sampling rate of 100 Hz, and we chose the EEG sleep signals from the Fpz-Cz channel for sleep stage classification analysis, since some studies revealed that EEG signals from Fpz-Cz and Pz-Oz can be replaced with each other without losing the AASM rules [31]. In this study, for every data set, we choose the sleep recording of nine hours from 11 p.m. to 8 a.m. to carry out the analysis.

### 2.2. Signal Preprocessing

**Figure 2:**
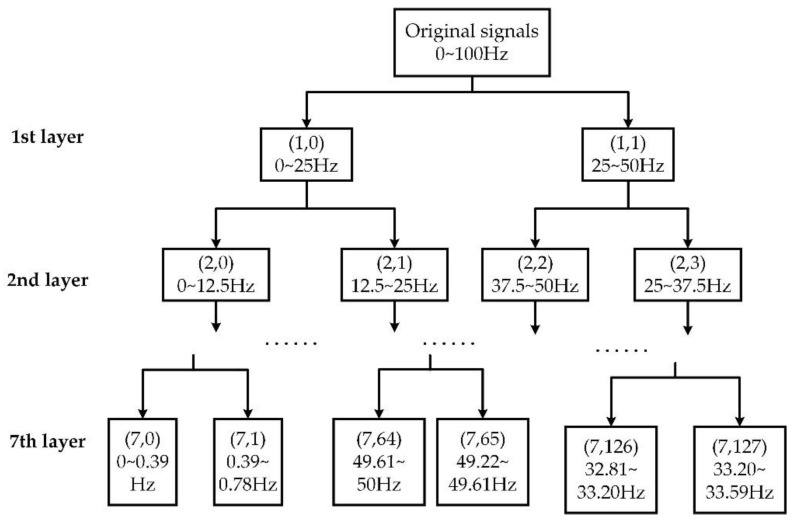
The wavelet packet tree applied for signal processing.

The WTD method was used to denoise the original EEG signals for signal preprocessing. Daubechies wavelets of the order 8 (db8) method were applied to decompose the collected EEG signals into 7 layers, as shown in Figure 2. After that, using the soft threshold method with suitable process coefficients, the EEG signals were denoised.

#### 2.2.1. Wavelet Packet Transform

EEG signals were decomposed into different characteristic waves [27,32], including alpha (α = (8–13 Hz)), beta (β = (12–30 Hz)), theta (θ = (4–8 Hz)), delta (δ = (0.5–2 Hz)), spindle (12–14 Hz), sawtooth (2–6 Hz), and K complex (1 Hz). The characteristic frequency waves corresponding to different stages were summarized and shown in Table 1. 

To obtain the characteristic waves from every denoised epoch, the WPT method was applied to obtain the corresponding frequency bands. The WPT method allows precisely resolving the brain rhythms into packets whilst demanding a relatively low computational cost [33,34]. To obtain all these characteristic waves, we constructed a wavelet packet tree with 7 decomposition levels to obtain the frequency band resolution of around 0.39 Hz. As seen in Figure 2, the wavelet packet coefficient of the *j*-th node in the *i*-th layer was named $\left(i,j\right),1\leq j\leq 2^{i}-1$, which represented a decomposed frequency band. We chose suitable nodes according to different characteristic waves’ frequency bands. By connecting these nodes’ frequency bands from low to high, a frequency band covering the required characteristic wave was formed. 

#### 2.2.2. Feature Extraction

Feature extraction was the essential process for accurate sleep stage classification. Since the sampling rate was 100 Hz in this study, there were a total of 3000 samples in each 30 s epoch. To obtain a comprehensive feature system, time-domain features, energy features, frequency-domain features, and nonlinear dynamics features were comprehensively considered in this study.

(1) Time-domain features

The standard deviation was the average amount of variability in each epoch. For each characteristic wave, the standard deviation of the samples during an epoch was computed as:

(1) $$Std=\sqrt{\overset{3000}{\underset{i=1}{\sum }}{\left(w_{i}-\bar{w}\right)}^{2}},$$

where $w_{i}$ was the *i*-th sample of an epoch corresponding to the characteristic wave. Thus, there were six standard deviations corresponding to six characteristic waves in an epoch: $Std_{\alpha },Std_{\beta },Std_{\delta },Std_{saw-tooth},Std_{\theta },Std_{spindle}$. Other effective time-domain features of the EEG signals were calculated and summarized (details in Appendix A.1, Table A1).

(2) Energy-domain features

The total energy of six characteristic waves in an epoch was defined as:

(2) $$Energy=\overset{3000}{\underset{i=1}{\sum }}w_{i}^{2},$$

Six energy features were corresponding to six characteristic waves in an epoch: $E_{\alpha },E_{\beta },E_{\delta },E_{saw-tooth},E_{\theta },E_{spindle}$. 

From previous research, N1 could not be classified accurately, which was usually considered to be confused with R or N2 [35]. To improve the accuracy of the N1 stage, two more different features were established in this study. Since the characteristic wave in the N1 stage was the θ wave, the characteristic waves for R and N2 were α and δ waves, respectively. Therefore, we set the ratio of the energy of alpha or delta to theta as important features:

(3) $$E_{\frac{\alpha }{\theta }}=\frac{E_{\alpha }}{E_{\theta }},\;$$

(4) $$E_{\frac{\delta }{\theta }}=\frac{E_{\delta }}{E_{\theta }},$$

where $E_{\alpha /\theta }$ in Equation (3) and $E_{\delta /\theta }$ in Equation (4) were the energy ratio of alpha and delta waves to theta wave, respectively.

(3) Frequency-domain features

**Figure A1:**
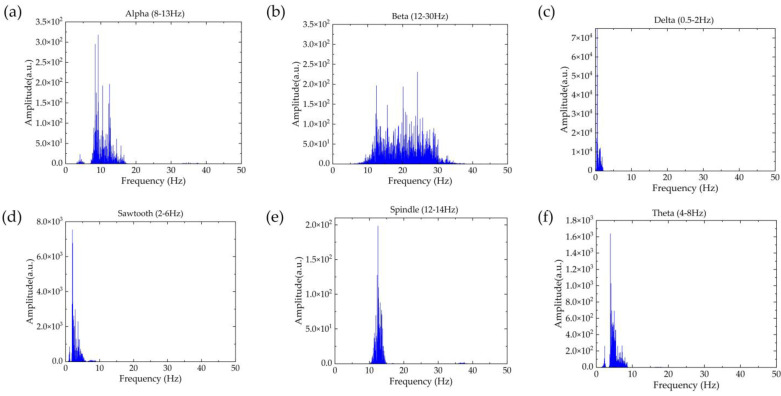
The frequency spectra of (**a**) Alpha, (**b**) Beta, (**c**) Delta, (**d**) Sawtooth, (**e**) Spindle, and (**f**) Theta, applied for computing frequency domain features.

The frequency features usually contain power information of EEG waves. In this study, the power of each characteristic wave was expressed as follows in Equation (5):

(5) $$Power=\sum_{k=1}^{K}P_{k},$$

where $P_{k}$ was the *k*th magnitude of the wave’s power spectral density (PSD), and K was the total sample number of the EEG signals in the frequency domain. Thus, six power features were corresponding to six characteristic waves (frequency spectra as in Figure A1) in an epoch: $P_{\alpha },\;P_{\beta },\;P_{\delta },\;P_{saw-tooth},\;P_{\theta },\;P_{spindle}$.

Moreover, mean frequency (*MNF*) for an epoch was used, which was defined as:

(6) $$MNF=\frac{\sum_{k}p_{k}f_{k}}{\sum_{k}p_{k}},$$

where $p_{k}$ and $f_{k}$ in Equation (6) were the *k*-th power and frequency of the power spectral density of the EEG signals in an epoch, respectively.

Similar to the energy features, we set the ratio of the power of alpha and delta to theta as new features:

(7) $$P_{\alpha /\theta }=\frac{P_{\alpha }}{P_{\theta }},P_{\delta /\theta }=\frac{P_{\delta }}{P_{\theta }},$$

where $P_{\alpha /\theta }$ and $P_{\delta /\theta }$ in Equation (7) were the power ratio of alpha and delta waves to theta wave, respectively.

(4) Nonlinear-dynamics-domain features

In this study, nonlinear-dynamics-domain features of Renyi entropy, Lempel–Ziv complexity, multi-scale entropy, spectral entropy, sample entropy, and fuzzy entropy were calculated with denoised EEG signals. 

Renyi entropy (*RE*) was widely applied to analyze EEG signals as well [36,37]. RE can quantify the diversity, uncertainty, or randomness of a system. *RE* values were calculated as in Equation (8):

(8) $$RE=-\log(\underset{k}{\sum }p_{k}{}^{2}),$$

Similarly, we calculated the *RE* values of six characteristic waves $\left(RE_{\alpha },\;RE_{\beta },\;RE_{\delta },\;RE_{saw-tooth},\;RE_{\theta },\;RE_{spindle}\right)$.

The variation in EEG signals within a time scope indicated the self-invariant and self-similar structures, and this was measured by the nonlinear analysis method of the Lempel–Ziv complexity (LZC) algorithm.

Before calculating the LZC values, the sequence $A^{n}$ was transformed into a finite symbol sequence, namely a binary sequence $Z=\left\{z_{1},z_{2},\ldots ,z_{n}\right\}$ as in Equation (9) with the threshold $T_{d}$:

(9) $$z_{i}=\{\begin{array}{c} 0,a_{i}<T_{d}\\ 1,otherwise \end{array}\;,$$

**Figure 3:**
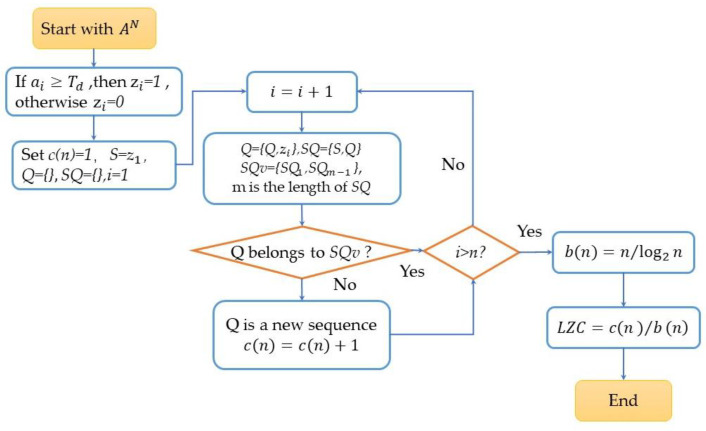
The nonlinear analysis computation process of Lempel–Ziv complexity (*LZC*).

The median of the sequence $A^{n}$ was taken as $T_{d}$. *LZC* was calculated following the computational flow chart as in Figure 3. 

Based on chaos theory, multi-scale entropy contributed to the improvement of the accuracy of sleep stage classification. We set the scale factor *τ*, and the sequence $A^{n}$ was divided into *τ* sequences. The coarsely granulated time sequence is given by $\lambda^{\tau }\left(J\right)=\left\{\lambda_{1}^{\tau },\lambda_{2}^{\tau },\ldots ,\lambda_{J}^{\tau }\right\}$:

(10) $$\lambda_{j}^{\left(\tau \right)}=\frac{1}{\tau }\sum_{i=\left(j-1\right)\tau +1}^{j\tau }a_{i},1\leq j\leq \left[\frac{n}{\tau }\right],$$

Considering the scale factor effect on the accuracy of sleep stage classification, R and N1 could not be classified properly if 1≤τ≤8. However, if τ≥14 W, R, and N2 stages would be confused, 9<τ<13 was therefore suitable. In this study, we set τ=11. Therefore, the multi-scale entropy in an epoch was expressed as the following:

(11) $$MsEn\left(m,r,\lambda^{\left(\tau \right)}\right)=\sum_{j=1}^{\left[\frac{n}{\tau }\right]}SpEn\left(m,r,\lambda^{\left(\tau \right)}\right),\tau =11,$$

where m=2, r=0.2 SD. Other nonlinear-dynamics-domain features of the EEG signals were calculated (details in Appendix A.2.).

#### 2.2.3. Feature Selection

**Figure 4:**
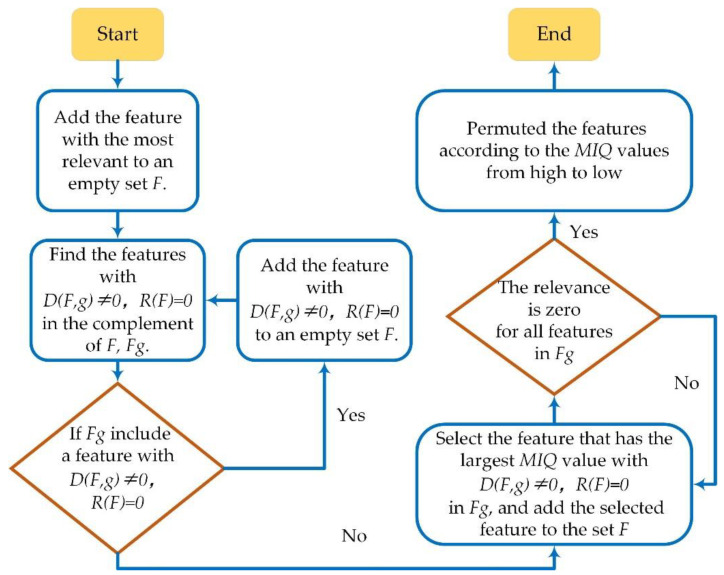
The computation process of mRMR to select the effective features.

In this article, the minimum redundancy maximum relevance (mRMR) algorithm was used to select the effective features. The mutual information *I* of the discrete random variables *Z*_1_ and *Z*_2_ was defined as [38] in Equation (12):

(12) $$I\left(Z_{1},Z_{2}\right)=\sum_{i,j}P\left(Z_{1}=Z_{1i},Z_{2}=Z_{2j}\right)log\frac{P\left(Z_{1}=Z_{1i},Z_{2}=Z_{2j}\right)}{P\left(Z_{1}=Z_{1i}\right)P\left(Z_{2}=Z_{2j}\right)}$$

where $P\left(Z_{1}=Z_{1i}\right)$, $P\left(Z_{2}=Z_{2j}\right)$, and $P\left(Z_{1}=Z_{1i},Z_{2}=Z_{2j}\right)$ were the probability density functions. The relevance between features was F. The output sequence $g=\left\{g_{1},g_{2},\ldots ,g_{end}\right\}$ was D(F,g) in Equation (13), and the redundancy in the feature sets *F* was R(F) in Equation (14), which were defined as:

(13) $$D\left(F,g\right)=\frac{1}{\left|F\right|}\sum_{f_{i}\epsilon F}I\left(f_{i},g\right),$$

(14) $$R\left(F\right)=\frac{1}{{\left|F\right|}^{2}}\underset{f_{i},f_{j}\epsilon F}{\sum }I\left(f_{i},f_{j}\right),\;$$

where $f_{i}$ and $\;f_{j}$ were different feature sets: $f_{i}=\left\{f_{i1},f_{i2},\ldots f_{i,end}\right\},f_{j}=\left\{f_{j1},f_{j2},\ldots f_{j,end}\right\}$. |*F*| was the number of the total features in *F*, which was 51 in this study (details in Appendix A.3, Table A2). The analysis process of the mRMR was shown in Figure 4. 

The *MIQ* value of each feature in feature set *F* was defined as in Equation (15):

(15) $$MIQ\left(f_{i}\right)=\frac{I\left(f_{i},g\right)}{\frac{1}{\left|F\right|}\sum_{f_{j}\in S}I\left(f_{i},f_{j}\right)},f_{i}\neq f_{j},$$

Finally, we obtained the *MIQ* values for all those features. The rank of *MIQ* values for different features was permuted (in Appendix A.4, Table A3).

### 2.3. Cascaded Support Vector Machine Classifier

The cascaded SVM method, consisting of two 3-class SVM models, was applied for the sleep stage classifier in this study. Since SVM is inherently a binary classifier, we chose the one-against-one method and constructed 3 hyper-planes, where each hyper-plane was constructed with the training epochs with two classes from three classes. To decide each epoch, the same voting weight was set for every decision function. Finally, the predicted result was the class with the largest vote.

#### 2.3.1. Data Set for SVM I

All these collected EEG signals were divided into 30 s epochs. Every epoch was assigned to one of the five sleep stages, which were W, N1, N2, N3, and R [39]. In our study, the proposed SVM I was applied to identify three different sleep stages: W, REM-LS, and N3. The REM-LS includes R, N1, and N2 stages, which are frequently confused with each other [27].

#### 2.3.2. Data Set for SVM II

Since the sample sizes of different sleep stages from the original data were different, as in Table 2, the N1 stage usually could not be classified accurately. To overcome this problem, we reconstructed the training set for the SVM II classifier from the training set of SVM I. Then, we collected total epochs in stage N1 and randomly selected the same number of epochs in stage R, and twice the number of epochs in stage N2 to make up the training data set. The training progress for the SVM II was conducted by 10-fold cross-validation with the formed training set, and the generalization performances of SVM I and SVM II were assessed by the same test set.

#### 2.3.3. Transform the Input Data

The training set was expressed as $Data={\left\{\left(X_{i}{}^{*},y_{i}\right)\right\}}_{i=1}^{M}$, where M was the number of total epochs in the training set. $X_{i}{}^{*}$ was an input vector for the *i*-th epoch: $X_{i}{}^{*}=\left[X_{i1}{}^{*},\ldots ,X_{i\chi }{}^{*}\right]$; $y_{i}$ was the label of the *i*-th epoch. For the SVM method, multiple classification problems were decomposed into several dichotomy problems. For each dichotomy problem, the label was $y_{i}\in \left\{1,-1\right\}$.

There were χ features and *M* epochs to form an input matrix *X*. The *k*-th element of the input matrix in the *i*-th epoch was standardized as in Equation (16):

(16) $$X_{ik}=\frac{X_{ik}{}^{*}-mean\left(X_{k}{}^{*}\right)}{std\left(X_{k}{}^{*}\right)},k=1,\ldots ,\chi ,$$

where $X_{k}{}^{*}$ was the vector for the *k*-th feature. 

The kernel function was applied to fulfill the space transform with a mapping relationship. In this study, the quadratic polynomial kernel function was utilized in Equation (17):

(17) $$\theta_{i}\theta_{j}=\kappa \left(X_{i},X_{j}\right)={\left(X_{i}{}^{T}\;X_{j}\right)}^{2},$$

$X_{i}$ and $X_{j}$ were different input vectors; $\theta_{i},\theta_{j}$ were the outputs in higher dimensional space corresponding to $X_{i},X_{j}$ through nonlinear mapping [40].

SVM constructed an optimal separating hyper-plane (OSH) by maximizing the margin of separation between the classes [40]. The separate hyper-plane was expressed as in Equation (18):

(18) $$W\theta +\varepsilon =0{\;\mathrm{such}\;\mathrm{that}\;y}_{i}\left(W\theta_{i}+\varepsilon \right)\geq 1\forall_{i},$$

where *W* was the normal plane and ε was the relative position to the coordinate center. We utilized a quadratic programming problem to find the OSH by creating a Lagrangian multiplier and converting it into the dual problem shown in Equations (19) and (20):

(19) $$\mathrm{maximize}\;\sum_{i=1}^{M}\alpha_{i}-\frac{1}{2}\sum_{i=1}^{M}\sum_{j=1}^{M}\alpha_{i}\alpha_{j}y_{i}y_{j}\kappa \left(X_{i},X_{j}\right),$$

(20) $$s.t.\overset{M}{\underset{i=1}{\sum }}\alpha_{i}y_{i}=0,\;0\leq \alpha_{i}\leq Const,\;$$

where $\;\alpha =\left(\alpha_{1},\ldots ,\alpha_{M}\right)$ was the Lagrangian multiplier, which was non-negative. *Const* was a constant regularization parameter. 

The final output $\overset{\wedge }{y_{j}}$ for the *j*-th epoch was expressed as in Equation (21):

(21) $$\overset{\wedge }{y_{j}}=\mathrm{sgn}(\sum_{i}^{M}\alpha_{i}y_{i}\kappa \left(X_{i},X_{j}\right)+\varepsilon ),$$

where $sgn(u)=\{\begin{array}{c} 1,u\geq 0\\ -1,u<0 \end{array}$.

We found that the number of input parameters was not linear with the accuracy of classification results. When the number of input parameters was small, the accuracy of classifiers increased very quickly. However, after a certain number of input parameters, the accuracy changed little. The proper number of input features was chosen to obtain a balance between the accuracy result and computing time.

## 3. Results and Discussion

### 3.1. The Average Accuracy of Sleep Stage Classification

In this study, we randomly selected 90% labeled epochs to train the SVM classifiers with 10% data reserved for the classification accuracy test. To test the accuracy of the trained model, different epochs were classified with SVM classifier I and classifier II, and then the classification results were compared with true labels to obtain the test accuracy. The training and testing were conducted five times to obtain five random training and testing sets. The average accuracy values were used to evaluate the generalization performance of the model.

Through the above process, we compared the average accuracy of the cascaded SVM model and the single SVM model, as summarized in Table 3. The average accuracy for the single SVM model was 86.45% and the standard deviation was around 0.71%. For the cascaded SVM model, the average accuracy was 88.11% and the standard deviation was around 0.67%. Additionally, for the cascaded SVM method, the accuracy of N1 was 55.65% and the standard deviation was 3.13%, while the value of the single SVM method was only 41.5% and the standard deviation was 1.72%. These results indicated that the cascaded SVM method was effective to improve the overall average classification accuracy. Moreover, the accuracy of the N1 stage was significantly improved. We found that it did not take too much time for the cascaded SVM to identify a sleep stage compared with the single SVM. All results in Table 3 were calculated by the computer with Intel (R) Core (TM) i9-9900K CPU @ 3.60 GHz, 32 GB of memory, and a 64-bit operating system based on a ×64 processor. The computing time was the average run time of the process from denoised EEG signals to give the sleep stage results.

### 3.2. The Sleep Stage Classification Performance

**Figure 5:**
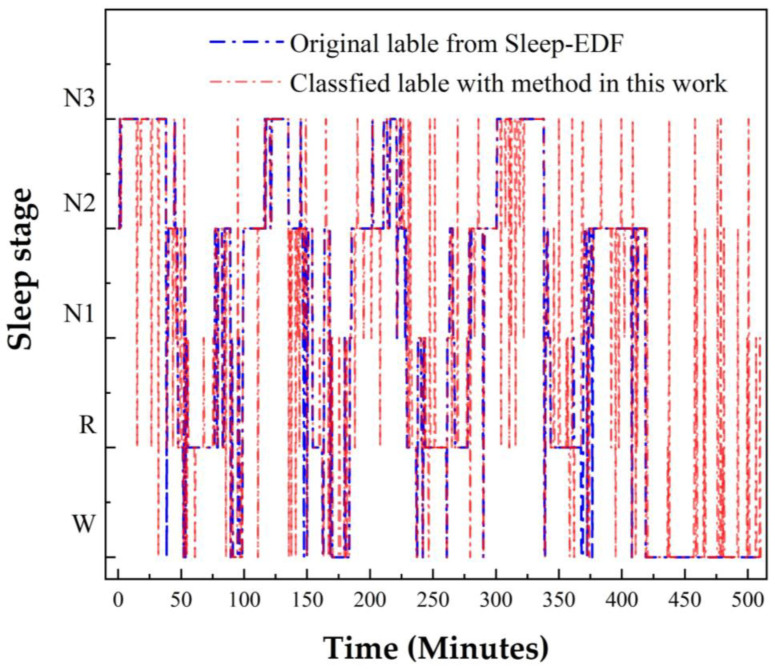
The classification performance was evaluated with the test set of SC4001.

To verify the performance of this proposed method for long-term applications, we compared the classification results with the method in this work and the original result from the Sleep-EDF data set (SC4001) as an example, shown in Figure 5. 

**Figure 6:**
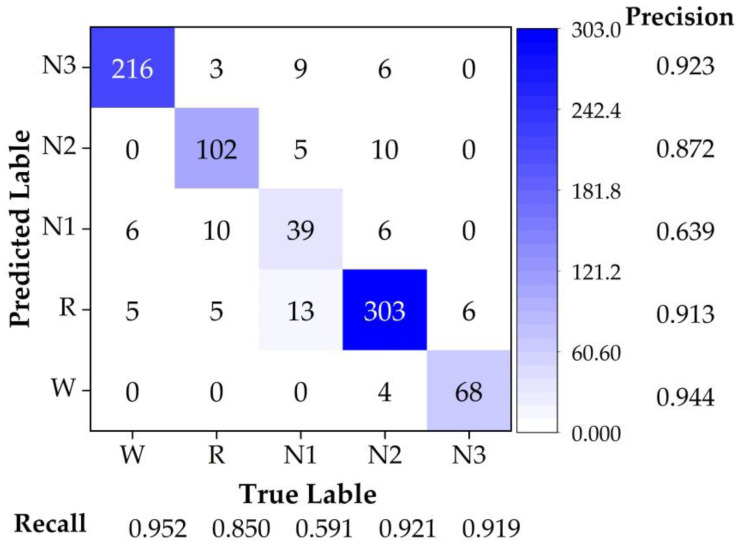
The confusion matrix of trained model was calculated with different stages for test set of SC4001.

The accuracy result for this case (SC4001) was 89.22%. It was clear that this method could be applied for continuous data analysis. To obtain more details about the classification result, we acquired the confusion matrix as shown in Figure 6. The most accurate stage was W, with a precision of 94.4%. In addition, due to the optimized model we acquired the precision of the N1 stage to be high, up to 63.9%. As in the confusion matrix, the total accuracy of this case was defined as in Equation (22):

(22) $$Acc_{i}=\frac{\sum N_{stagei,PT}}{\sum N_{stagei,PT}+\sum N_{stagei,F}},$$

where $Acc_{i}$ is the total accuracy of the model; $N_{stagei,PT}$ is the number of corrected predicted samples for sleep stage *i* (W, R, N1, N2, and N3); and $N_{stagei,F}$ is the number of prediction and true label mismatched samples for sleep stage *i*.

The precision of every labeled stage by prediction *Pi* was defined as in Equation (23):

(23) $$Precition_{Pi}=\frac{N_{stagePi,PT}}{N_{stagePi,PT}+\sum N_{stagePi,F}},$$

where $N_{stagePi,PT}$ is the number of corrected predicted samples for the predicted labeled of stage *Pi* in the matrix; $N_{stagePi,PT}$ is the number of prediction and true label mismatched samples for stage *Pi* in the matrix.

The recall of every original labeled stage *Ti* was defined as in Equation (24):

(24) $$Recall_{Ti}=\frac{N_{stageTi,PT}}{N_{stageTi,PT}+\sum N_{stageTi,F}},$$

where $N_{stageTi,PT}$ is the number of corrected predicted samples for the originally labeled stage of *Ti* in the matrix. $N_{stagePi,PT}$ is the number of prediction and true label mismatched samples for stage *Ti* in the matrix.

In addition, the classification accuracy results using the cascaded Support Vector Machine method in this study and other previously reported studies are summarized in Table 4. 

In Table 4, these methods classifying the five sleep stages, which were W, N1, N2, N3, and R, were compared. The prediction accuracy of Neural Networks is usually higher than that of SVM models, but large amounts of data are needed for calculation using Neural Networks. However, for the sleep stage classification task with single-channel EEG, we considered a more comprehensive feature system including the time-domain, energy-domain, frequency-domain, and nonlinear-dynamics-domain features to obtain better results in this work. Moreover, for N1 stage classification, we not only chose the special frequency features but also increased the number of N1 stages in the training set to acquire the model. From the application perspective, the cascaded Support Vector Machine method applied in our study can be applied for the EEG signal analysis for various applications. Based on the results presented above, it was demonstrated that our method can classify the sleep stage in a short time, which shows the potential for long-term sleep stage monitoring.

### 3.3. Influence by Number of Input Features on Classification Accuracy

**Figure 7:**
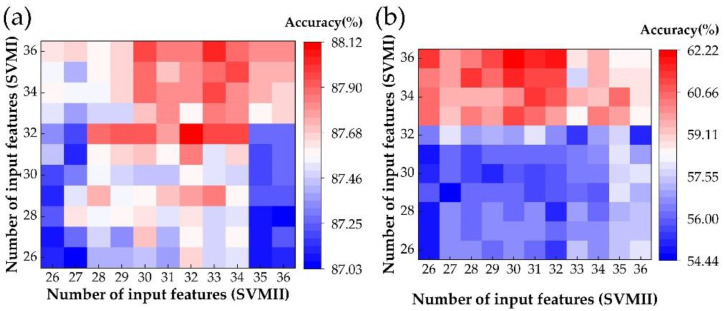
The average accuracy of five sleep stages’ classifications (**a**) and accuracy of sleep stage N1 (**b**), with the different number of input features.

Based on our preliminary calculation results, with the number of classifier inputs increasing, the accuracy of classifiers would increase very quickly at first. However, after a certain number of input parameters, there was little change in accuracy. To investigate how the number of input features affects classification accuracy, we took the first 26 to 36 features from the permuted rank of 51 features as the input features for every SVM model to calculate the mean accuracy of the total task, as in Figure 7a. Moreover, the accuracy of the N1 stage was also calculated, as shown in Figure 7b. As in Figure 7a, the accuracy of SVMI increased slightly when the number of input features was larger than 32, while a similar phenomenon was found for SVMII when the number of input features was larger than 30. Based on these calculation results, we chose 32 and 30 as the number of input features for calculation.

We found that the most suitable number of input features can be chosen without affecting the classification accuracy.

### 3.4. Effectiveness Analysis of Different Nonlinear Dynamics Features

In this study, fuzzy entropy, LZC, sample entropy, and multi-scale entropy needed more time to be calculated than other features, whereas they contributed conspicuously to the improvement of accuracy. A fast calculation model needs to evaluate the computing time, average accuracy value of five sleep stages, and the accuracy value of stage N1 simultaneously to compare them. The technique for order preference by similarity to an ideal solution (TOPSIS) method was applied to give a comprehensive evaluation of every nonlinear dynamic feature. With TOPSIS test results, suitable nonlinear dynamics features were selected.

We set *j*, $Acc_{j}$, and $AccN1_{j}$ to represent the computing time, average accuracy value, and the accuracy value of N1 for the *j*-th nonlinear dynamics feature before normalized operation. For the normalized operation, it was defined as in Equation (25):

(25) $$\{\begin{array}{c} V_{j1}=\frac{\max\{Time\}-Time_{j}}{\max\{Time\}-\min\{Time\}}\\ V_{j2}=\frac{Acc_{j}-\min\{Acc\}}{\max\{Acc\}-\min\{Acc\}}\\ V_{j3}=\frac{AccN1_{j}-\min\{AccN1\}}{\max\{AccN1\}-\min\{AccN1\}} \end{array}j=1,2,3,4,$$

where $V_{j1},V_{j2},V_{j3}$ were the computing time, average accuracy for five sleep stages, and the accuracy of N1 with the normalized operation.

Then, we set the positive and negative ideal values for each parameter: $V_{i}{}^{+},V_{i}{}^{-},i=1,2,3$. The distances to the positive and negative ideal solutions were solving using Equations (26) and (27):

(26) $$D_{j}{}^{+}=\sqrt{\sum_{i=1}^{3}\alpha_{i}{\left(V_{ji}-V_{i}{}^{+}\right)}^{2}}$$

(27) $$D_{j}{}^{-}=\sqrt{\sum_{i=1}^{3}\alpha_{i}{\left(V_{ji}-V_{i}{}^{-}\right)}^{2}}$$

where $D_{j}{}^{+}$, $D_{j}{}^{-}$ represented the distances to the positive and negative ideal solutions, respectively, and $\alpha_{1},\alpha_{2},\alpha_{3}\;$ were the weight of these features. Considering the equal importance of computing time and accuracy value, we took $\alpha_{1}=\alpha_{2}=\alpha_{3}=1/3$.

After that, the comprehensive score for the *j*-th feature was defined as in [41] with Equation (28):

(28) $$score_{j}=\frac{D_{j}{}^{-}}{D_{j}{}^{+}+D_{j}{}^{-}},j=1,2,3,4,$$

All these scores of every parameter are shown in Table 5. We found that different kinds of nonlinear dynamics features have different influences on the average accuracy results. The impact on the accuracy results of the multi-scale entropy feature was the most significant, even though the computing time of this kind of feature was the longest. In a real application, to obtain a balance between the accuracy results and the computing time the most significant features can be chosen without other kinds of features. In this study, it was clear that multi-scale entropy was more suitable to be selected. For EEG signals from a single channel, it was very complex to acquire a precise description. The multi-scale entropy described the single complexity, which was efficient for sleep stage classification. 

Through the above-mentioned process, the overall performances of the model before and after the selection of nonlinear dynamics features were obtained and are shown in Table 6. 

With all these nonlinear features included, the average computing time for each epoch was longer than 2.5 s. After nonlinear dynamic features selection, the computing time was 1.65 s, which was much shorter with the accuracy maintained. Nonlinear dynamic features have a great influence on the performance of the sleep stage classification model. 

## 4. Conclusions

In this work, a fast sleep stage classification applicable method with energy, time, frequency, and nonlinear dynamics features of EEG signals from the Fpz-Cz channel with a cascaded Support Vector Machine was proposed. Compared with the traditional single SVM model, the average accuracy of the N1 stage was enhanced by up to 55.65%. Moreover, different kinds of nonlinear dynamics features were included in the model, and it was revealed that different nonlinear dynamics features had different effects on the performance of the model. The feature of multi-scale entropy was the most significate parameter. To achieve a balance between accurate and time-consuming classification, we selected nonlinear dynamics features based on overall performance parameters. Finally, the proposed method was shown to be fast, with a classification time of less than 1.7 s, and real applicable sleep stage classification with a high accuracy of 88.11%. Based on the proposed method, further investigations could be conducted with multi-channel EEG signal analysis for other applications. Moreover, signal preprocessing and feature extraction methods were explored to further decrease the time taken for classification and increase the generalization performance. For real applications for long-term sleep medical diagnosis exploration, the robustness of this method can be further enhanced with more samples for every user.

## 5. Patents

The patent resulting from the work reported in this manuscript is CN202111182428.9.

## Figures and Tables

**Table 1:** Five sleep stages and their corresponding characteristic frequency.

Sleep Stage	Characteristic Waves
W	Alpha (8–13 Hz) and beta (12–30 Hz)
N1	Theta (4–8 Hz)
N2	Spindle (12–14 Hz) and K complex (1 Hz)
N3	Delta (0.5–2 Hz)
R	Alpha (8–13 Hz), beta (12–30 Hz), theta (4–8 Hz), and sawtooth wave (2–6 Hz)

**Table 2:** The sample size distribution in different sleep stages.

Sleep Stage	Number of Epochs in Stages	Proportion
W	2201	26.97%
N1	579	7.10%
N2	3221	39.47%
N3	900	11.03%
R	1259	15.43%

**Table 3:** The run time of training and testing of the Support Vector Machine for each epoch.

	Cascaded SVM	SVM
Computing time for each epoch (s)	1.65	1.57
Average accuracy	88.11% ± 0. 67%	86.45% ± 0.71%
The average accuracy of N1	55.65% ± 3.13%	41.5% ± 1.72%

**Table 4:** The accuracy of the classification for five sleep stages using single-channel methods.

Reference	Classifier	Accuracy	Accuracy of N1
[41]	OCRNN	82.40%	33.39%
[18]	LSTM RNN	86.74%	61.09%
[42]	Elman RNN	87.20%	36.70%
[43]	Bagging	86.53%	27.48%
[26]	CNN	86.79%	34.92%
[44]	Multi-class SVM	83.92%	17.39%
This work	Cascaded Support Vector Machine	88.11%	55.65%

**Table 5:** The evaluation of nonlinear dynamics features.

Features	Computing Time(s)	Accuracy	Accuracy of N1	Score
Fuzzy entropy	2.04 ± 0.012	0.8560 ± 0.0133	0.4685 ± 0.0638	0.6136
LZC	2.03 ± 0.015	0.8586 ± 0.0143	0.4550 ± 0.0647	0.5399
Sample entropy	2.04 ± 0.021	0.8589 ± 0.0096	0.4750 ± 0.0740	0.3808
Multi-scale entropy	2.02 ± 0.014	0.8651 ± 0.0152	0.4524 ± 0.0391	0.5858

**Table 6:** The performance of different models.

Performance Parameter	Before Nonlinear Features Selection	After Nonlinear Feature Selection	Total Features
Computing time for each epoch (s)	2.08	1.65	2.65
Average accuracy	74.54% ± 0. 82%	88.11% ± 0.67%	74.36% ± 1.93%
Average accuracy of N1	26.58% ± 1.76%	55.65% ± 3.13%	26.97% ± 0. 75%

## Data Availability

The data presented in this study are openly available in Physionet: https://physionet.org/content/sleep-edfx/1.0.0/sleep-cassette/ (accessed on 11 November 2020).

## Appendix A

### Appendix A.1. Effective Time-Domain Features of the EEG Signals

**Table A1:** Functions of time-domain features ***^1^**.

Time-Domain Features	Functions
Mean	$\bar{x}=\frac{1}{3000}\overset{3000}{\underset{i=1}{\sum }}x_{i}$
Median	$M=\frac{x_{1500}+x_{1501}}{2}$
Variance	$var(x)=\frac{1}{3000}\overset{3000}{\underset{i=1}{\sum }}{\left(x_{i}-\bar{x}\right)}^{2}$
Skewness	$skew\left(x\right)=\frac{\frac{1}{3000}\sum_{i=1}^{3000}{\left(x_{i}-\bar{x}\right)}^{3}}{var{\left(x\right)}^{3/2}}$
Kurtosis	$kurt\left(x\right)=\frac{\frac{1}{3000}\sum_{i=1}^{3000}{\left(x_{i}-\bar{x}\right)}^{4}}{var{\left(x\right)}^{2}}$
Hjorth mobility	$HM\left(x\right)=\sqrt{\frac{var(dx/dt)}{var(x)}}$
Peak-to-peak amplitude	$x_{ppk}=max(x)-min(x)$
Hjorth complexity	$HC\left(x\right)=\frac{HM\left(dx/dt\right)}{HM\left(x\right)}$
Average rectified value	$ARV\left(x\right)=\frac{1}{3000}\overset{3000}{\underset{i=1}{\sum }}\left|x_{i}\right|$
Root mean square	$RMS\left(x\right)=\sqrt{\frac{1}{3000}\overset{3000}{\underset{i=1}{\sum }}|x_{i}{|}^{2}}$

***^1^** In this table, $x_{i}$ and $\bar{x}$ were the value of the *i*th sample and the mean value of an epoch for denoised EEG signals, respectively.

### Appendix A.2. Nonlinear-Dynamics-Domain Features of the EEG Signals

(1) Spectral entropy (SE)

Spectral entropy (SE) is a nonlinear method to summarize signal power irregularity over measured frequencies, which describes the complexity of a system [45], which is widely used to analyze the electrophysiological signal [46,47]. It can be defined as the following:

(A1) $$SE=\underset{k}{\sum }p_{k}\log(\frac{1}{p_{k}})$$

where $p_{k}$ is the *k* th power spectral density of the EEG signals in an epoch. The spectral entropy of six characteristic waves ($SE_{\alpha },SE_{\beta },SE_{\delta },SE_{saw-tooth},SE_{\theta },SE_{spindle}$) were calculated in this study.

(2) Sample entropy

Sample entropy measures the complexity of the time series by measuring the probability of new patterns being generated in the signals. Meanwhile, if the probability of new patterns being generated is higher, the complexity of the signal is higher as well [48]. It is also suitable to analyze EEG signals with low signal noise [49]. To calculate the sample entropy of EEG signals in an epoch, we suppose the length of samples is N and a discrete time sequence is $A^{n}=\left\{a_{1},a_{2},\ldots ,a_{n}\right\}$, and define the m-dimensional vector in $A^{n}$ [50] as the following:

(A2) $$A_{m}\left(i\right)=\left\{a_{i},a_{i+1},\ldots ,a_{i+m-1}\right\},i=1\ldots \ldots ,n-m$$

In this article, n=3000. The distance between $A_{m}\left(i\right)$ and $A_{m}\left(j\right)$ can be defined as the following:

(A3) $$d_{ij}^{m}=d\left[A_{m}\left(i\right),A_{m}\left(j\right)\right]=\underset{k=0,\ldots ,m-1}{max}\left(\left|a_{i+k}-a_{j+k}\right|\right)$$

The next step is to obtain the probability of distance smaller than the similarity tolerance *r* as the following:

(A4) $$B^{m}\left(r\right)=\frac{\sum_{i=1}^{n-m}B_{i}}{N-m-1}$$

where $B_{i}$ is the number of the $d_{ij}^{m}$ ($d_{ij}^{m}<r$), *r* is effective when it satisfies r∈[0.1SD,0.25SD], and *SD* is the standard deviation of the sequence $A^{n}$. We took m=2, r=0.2SD. Then, we increased the dimension from *m* to *m +* 1 and repeated the above steps to obtain the sample entropy value:

(A5) $$SpEn\left(m,r\right)=\underset{n\to \infty }{lim}\{-\ln[\frac{B^{m+1}\left(r\right)}{B^{m}\left(r\right)}]\}$$

(3) Fuzzy entropy

Apart from the sample entropy, fuzzy entropy should be calculated, which shows the probability of new patterns being generated in the signals [51]. To obtain that value, we acquired the degree of the membership $\mu_{ij}^{m}$.

(A6) $$\mu_{ij}^{m}=\{\begin{array}{c} \exp[-\left(\frac{d_{ij}^{m}}{r}{)}^{2}\ln2\right],d_{ij}^{m}=0\\ 1,d_{ij}^{m}>0 \end{array}$$

where m=2, r=0.15SD, and $d_{ij}^{m}$ is the distance between $A_{m}\left(i\right)$ and $A_{m}\left(j\right)$. Define $\varphi^{m}\left(r\right)=\frac{1}{n-m+1}\overset{n-m+1}{\underset{i,j}{\sum }}\mu_{ij}^{m}$, and the fuzzy entropy is the following:

(A7) $$FzEn\left(m,r,s\right)=-\ln\frac{\varphi^{m}\left(r\right)}{\varphi^{m+1}\left(r\right)}$$

### Appendix A.3. The Feature System Considered in this Study

**Table A2:** Different features considered in this study.

Time-Domain Features	Energy-Domain Features	Frequency-Domain Features	Nonlinear-Dynamics-Domain Features
$1\sim 6:Std_{\alpha },Std_{\beta },Std_{\delta },$ $Std_{saw-tooth},Std_{\theta },Std_{spindle}$	$17\sim 22:E_{\alpha },E_{\beta },E_{\delta },$ $E_{saw-tooth},E_{\theta },E_{spindle}$	$\begin{array}{l} 25\sim 30:P_{\alpha },P_{\beta },P_{\delta },\\ P_{saw-tooth},P_{\theta },P_{spindle} \end{array}$	$34\sim 39:SE_{\alpha },SE_{\beta },SE_{\delta },$ $SE_{saw-tooth},SE_{\theta },SE_{spindle}$ $40\sim 45:RE_{\alpha },RE_{\beta },RE_{\delta },$ $RE_{saw-tooth},RE_{\theta },RE_{spindle}$ 46: Spectral entropy
7: Mean	$23:E_{\frac{\alpha }{\theta }}=\frac{E_{\alpha }}{E_{\theta }}$	31: Mean frequency	47: Renyi entropy
8: Median	$24:E_{\frac{\delta }{\theta }}=\frac{E_{\delta }}{E_{\theta }}$	$32:P_{\frac{\alpha }{\theta }}=\frac{P_{\alpha }}{P_{\theta }}$	48: Sample entropy
9: Variance		$33:P_{\frac{\delta }{\theta }}=\frac{P_{\delta }}{P_{\theta }}$	49: Fuzzy entropy
10: Skewness			50: Multi-scale entropy
11: Kurtosis			51:Lempel–Ziv complexity
12: Hjorth mobility			
13:peak-to-peak amplitude			
14: Hjorth complexity			
15: Average rectified value			
16: Root mean square (RMS)			

### Appendix A.4. The Permuted Rank of Different Features for SVM Classifiers

There are ranks shown in Table A3, which are permuted according to the MIQ value from high to low.

**Table A3:** The ranks of effective features for different classifiers.

Classifier	Ranks of Features’ Number
Support Vector Machine I	46→49→11→40→10→24→32→50→20→7 12→33→35→47→8→29→13→51→42→15 25→31→43→26→3→48→14→44→9→37 36→41→23 →34→16→27→4→2→38→17 19→28→5→18→1→21→39→45→6→22→30
Support Vector Machine II	20→7→24→2→32→33→17→11→19→39 43→13→49→21→45→50→14→37→40→30 15→31→35→44→4→36→18→25→9→23 28→48→41→34→42→6→38→12→3→26 1→46→5→16→22→27→51→29→47→8→10

In Table A3, the corresponding relations between features and numbers are shown in Table A2.
